# Bisphosphonate-associated osteonecrosis of the jaw is linked to suppressed TGFβ1-signaling and increased Galectin-3 expression: A histological study on biopsies

**DOI:** 10.1186/1479-5876-9-102

**Published:** 2011-07-04

**Authors:** Falk Wehrhan, Peter Hyckel, Arndt Guentsch, Emeka Nkenke, Phillip Stockmann, Karl A Schlegel, Friedrich W Neukam, Kerstin Amann

**Affiliations:** 1 Department of Oral and Maxillofacial Surgery University of Erlangen-Nuremberg, Germany; 2 Department of Plastic Surgery/St. Georg-Hospital Eisenach University of Jena, Germany; 3 Department of Conservative Dentistry University of Jena, Germany; 4 Institute of Pathology University of Erlangen-Nuremberg, Germany

**Keywords:** BRONJ, oral soft tissue, transforming growth factor beta 1, galectin-3, oral surgery

## Abstract

**Background:**

Bisphosphonate associated osteonecrosis of the jaw (BRONJ) implies an impairment in oral hard- and soft tissue repair. An understanding of the signal transduction alterations involved can inform therapeutic strategies. Transforming growth factor β1 (TGFβ1) is a critical regulator of tissue repair; galectin-3 mediates tissue differentiation and specifically modulates periodontopathic bacterial infection. The aim of this study was to compare the expression of TGFβ1-related signaling molecules and Galectin-3 in BRONJ-affected and healthy mucosal tissues. To discriminate between BRONJ-specific impairments in TGFβ1 signaling and secondary inflammatory changes, the results were compared to the expression of TGFβ1 and Galectin-3 in mucosal tissues with osteoradionecrosis.

**Methods:**

Oral mucosal tissue samples with histologically-confirmed BRONJ (n = 20), osteoradionecrosis (n = 20), and no lesions (normal, n = 20) were processed for immunohistochemistry. Automated staining with an alkaline phosphatase-anti-alkaline phosphatase kit was used to detect TGFβ1, Smad-2/3, Smad-7, and Galectin-3. We semiquantitatively assessed the ratio of stained cells/total number of cells (labeling index, Bonferroni-adjustment).

**Results:**

TGFβ1 and Smad-2/3 were significantly decreased (p < 0.032 and p(0.028, respectively) in the BRONJ samples and significantly increased (p < 0.04 and p <0.043, respectively) in the osteoradionecrosis samples compared to normal tissue. Smad-7 was significantly increased (p < 0.031) in the BRONJ group and significantly decreased (p < 0.026) in the osteoradionecrosis group. Galectin-3 staining was significantly (p < 0.025) increased in both the BRONJ and the osteoradionecrosis (p < 0.038) groups compared to the normal tissue group. However, Galectin-3 expression was significantly higher in the BRONJ samples than in the osteoradionecrosis samples (p < 0.044).

**Conclusion:**

Our results showed that disrupted TGFβ1 signaling was associated with delayed periodontal repair in BRONJ samples. The findings also indicated that impairments in TGFβ1-signaling were different in BRONJ compared to osteoradionecrosis. BRONJ appeared to be associated with increased terminal osseous differentiation and decreased soft tissue proliferation. The increase in Galectin-3 reflected the increase in osseous differentiation of mucoperiosteal progenitors, and this might explain the inflammatory anergy observed in BRONJ-affected soft tissues. The results substantiated the clinical success of treating BRONJ with sequestrectomy, followed by strict mucosa closure. BRONJ can be further elucidated by investigating the specific intraoral osteoimmunologic status.

## Introduction

Numerous attempts have been made to explain the development of bisphosphonate-associated osteonecrosis of the jaw (BRONJ), but the formal pathology remains unknown [1]. Previous studies have described the concordance of local BRONJ and an inflammatory reaction that was induced by an intraoral, gram-negative bacteria superinfection of the tissue [1,2]. Alternatively, there is increasing evidence that BRONJ is caused by bisphosphonate (BP)-related impairment of the interplay among osteoblasts, osteoclasts, fibroblasts, and keratinocytes during tissue remodeling. However, it remains unclear whether BRONJ arises from a laceration in the oral mucosa or from the underlying jaw bone tissue [1]. Recently, BRONJ was related to an impairment in Msx-1-related osteoblast proliferation [3]. However, results are contradictory regarding the biologic impact of BP on periodontal epithelial and connective tissue cells. BP gel formulations, topically applied in periodontal lesions, have not caused adverse effects [4]. In contrast, when alendronate tablets were held under a denture in contact with the oral mucosa, necrosis occurred [5]. BP was shown to stimulate bone progenitor cells toward osteogenesis *in vitro *[6]. In addition, the administration of zoledronic acid to oral gingival fibroblasts *in vitro *reduced expression of extracellular matrix (ECM) proteins, including collagens I, II, and III [7].

Transforming growth factor β1 (TGFβ1) is a pleiotropic cytokine that mediates fibroblast differentiation and proliferation and regulates the epithelial-to-mesenchymal transition (EMT) during wound repair {Huminiecki, 2009 #3987}. TGFβ1 exerts its intracellular actions through Smad protein signaling. Smad 2/3 was identified as the downstream TGFβ1 effector, and Smad 7 inhibited intracellular TGFβ1-related signaling [8]. Increased TGFβ1 and Smad-2/3 expression was shown to be related to fibrocontractive wound healing disorders [9]. Loss of TGFβ1 has been implicated in delayed wound healing and impaired ECM deposition [10]. TGFβ1 was shown to differentially affect epithelial and fibrous connective tissues; it inhibited the migration of epithelial cells during wound healing, but stimulated proliferation of fibroblasts [11] TGFβ1 and Smad signaling were shown to be involved in both osseous and connective tissue remodeling; thus, BP-related alterations in TGFβ1 signaling might explain BP-associated changes in the oral mucosa tissues of BRONJ affected jaws {Wu, 2009 #3990}. Furthermore, osteoradionecrosis has been associated with increased TGFβ1 expression [12].

BP-related changes in Smad-2/3 expression may also affect Smad activation by the glycoprotein, Galectin-3, in a TGFβ1-independent pathway [13]. Galectin-3 is involved in the regulation of epithelial and bone differentiation and plays a pivotal role in inflammatory responses and fibrotic tissue remodeling; it has been shown to inhibit the activation of cytokines by periodontopathic gram negative bacteria [14-16]. Galectin-3 expression was increased in radiation-impaired epithelial tissues. Galctin-3 expression in squamous epithelial tissues was positively associated with differentiation and negatively associated with proliferation {Szabo, 2009 #4519}. Therefore, the roles of TGFβ1 and Galectin-3 in cellular differentiation, tissue regeneration, and inflammation may be relevant to the mechanisms underlying BRONJ.

The American Society for Bone and Mineral Research has formed a BRONJ-task force that requires clinical and basic research in jaw-specific biology [17]. This study aimed to compare the cellular expression levels of TGFβ1, Smad 2/3, Smad 7, and Galectin 3 in BRONJ-related periodontal tissues compared to healthy oral mucosa. We assessed the impact of BP-therapy on the spatial distribution and protein expression of TGFβ1 signaling molecules and Galectin-3 in BRONJ sites with semiquantitative immunohistochemical analysis. To discriminate between BRONJ-specific impairments and secondary inflammatory changes that could affect TGFβ1 signaling, the results were compared to the expression of TGFβ1 and Galectin-3 in mucosal tissues with osteoradionecrosis.

## Materials and methods

### Patients and tissue harvesting

Oral mucosa specimens from 60 patients were included in this study. Twenty specimens were obtained from 20 consecutive patients with clinically and histologically evident BRONJ that underwent radical sequestrectomy. The ethical aspects of the study were approved by the ethical committee of the University of Erlangen-Nuremberg (Ref.-Nr. 4272). The specimens used in this study were from tissue samples collected for routine histopathologic diagnostics. Each specimen included was confirmed to exhibit histopathologic aspects of BRONJ. In addition to the histopathologic characteristics of BRONJ, the inclusion criteria for specimens were: patients received intravenous application of either pamidronate or zoledronate for at least 12 months for treating carcinoma, and patients showed clinical evidence of an exposed jaw bone for at least 8 weeks. Specimens from patients with former radiotherapy were excluded. The clinical data and the description of treatment procedures for the patients included in this study were documented previously [18]. All specimens were obtained during routine clinical procedures, where tissue was collected for standard diagnostics. Thus, no surgical procedure specific to this study was performed, and no additional material was collected from patients.

The controls comprised 20 alveolar mucosal specimens that were collected during intraoral surgery procedures in patients with no BP-history and no clinical signs of intraoral inflammation or periodontitis. Of the 20 control samples, 13 specimens were from the alveolar crest after a tooth extraction that required the removal of sharp bone ridges and adaptation of soft tissues; 4 specimens were from mucoperiosteal tissue extracted during orthognatic surgery in the lower jaw; 3 specimens were from mucoperiosteal tissue that covered wisdom teeth that required removal from the lower jaw. The gender and age of patients were matched in the BRONJ and control groups, except the 4 samples from the orthognatic surgery procedure. The average age of the patients in the BRONJ group was higher than that in the 4 normal patients that underwent orthognatic surgery.

The osteoradionecrosis specimens (n = 20) were from patients that had been treated with radiotherapy prior to surgery for oral squamous epithelial carcinoma. These patients received a mean total reference dose of 68 Gy in the lower jaw region. The specimens used in this study were collected after a mean interval of 36 months between radiotherapy and secondary surgery. Tissue samples were obtained from the soft tissue that surrounded the bone that was exposed during a sequestrectomy of osteoradionecrosis-affected mandibular bone. The osteoradionecrosis group consisted of 12 males and 8 females with a median age of 57 years. The 60 specimens used in this study were measured (average size: 5 × 3 × 3 mm) and then immediately fixed in 4% formalin.

### Immunohistochemical staining

The formalin-fixed, paraffin-embedded tissue samples were sliced in consecutive sections with a microtome (Leica, Nussloch, Germany) and then dewaxed in graded alcohol in preparation for immunohistochemical staining. Immunohistochemical staining was performed with the alkaline phosphatase-anti-alkaline phosphatase method and an automated staining device (Autostainer plus, DakoCytomation, Hamburg, Germany). We used the standard protocol recommended for the staining kit (Dako Real, Cat. K5005, DakoCytomation). Proteins were detected by incubating tissues in the autostainer (20°C, 1 h) with specific antibodies. TGFβ1 was detected with a polyclonal rabbit-IgG anti-human TGFβ1 antibody (anti-TGFβ1; sc-146, Santa-Cruz, Santa Cruz, USA; dilution 1:100). Smad-2/3 was detected with a polyclonal goat-IgG (anti-human Smad-2/3, sc-6033, Santa Cruz, USA; dilution: 1:100). Smad-7 was detected with a polyclonal goat-anti-human antibody (sc-9183, Santa Cruz, dilution 1:100). Galectin-3 was detected with a polyclonal rabbit-anti-human antibody (sc-20157, Santa Cruz, dilution 1:100). The secondary antibodies were included in the staining kit; biotinylated polyclonal, goat-anti-rabbit was used for TGFβ1 and Galectin-3; rabbit anti-goat IgG was used for Smad-2/3 and Smad-7 (E 0466, DAKO, dilutions 1:100). Stains were visualized with the Fast Red Solution, localized by biotin-associated activation of the secondary antibodies (ChemMate-Kit, Dako). This was followed by incubation in hematoxylin for counterstaining the nucleus. Two consecutive tissue samples were processed per immunohistochemical stain; one served as a negative control in each case (identical treatment, but replacement of the primary antibody with an IgG-istotype of the primary antibody). A positive control sample that was known to stain positive for a given antibody was included in each series.

### Semiquantitative immunohistochemical analysis

The BRONJ-related and healthy oral mucosa sections were examined qualitatively under a bright-field microscope (Axioskop, Zeiss, Jena, Germany) at 100-400 × magnification for differences in numbers and localization of stained mucosa cells, which comprised fibroblasts, fibrocytes, and periosteal progenitor cells. In the healthy samples, subepithelial tissues were examined, including connective, submucous, and epiperiosteal structures. Bone tissue was excluded from the analysis. In BRONJ samples, soft tissues attached to the necrotic zone were examined. For each sample, three visual fields per section were digitized at 200 × magnification with a CCD camera (Axiocam 5, Zeiss, Jena, Germany) and the Axiovision program (Axiovision, Zeiss, Jena, Germany). The digitized images were 800 × 500 μm at the original 200 × magnification. Randomized, systematic subsampling was performed based on the method of Weibel [19-22]. A semiquantitative analysis was performed to determine the cytoplasmic expression levels of TGFβ1, Smad-2/3, Smad-7, and Galectin-3. The labeling index was defined as the percentage of expressing cells (ratio of positively stained cells to the total number of cells per visual field, multiplied by 100). Cells of fibroblast lineage, including perisoteal progenitor cells, were recognized by their spindle shape. Endothelial cells and epithelial cells were excluded from counting. Cell counting was performed by 3 independent observers that were not engaged in the project; all were familiar with tissue morphology analyses and immunohistochemical methods. The observers were blinded to the tissue origin of the visual fields. The qualifaction of the 3 observers were dentist (1) and physician (2) engaged in their dental/medical thesis dealing with signal transduction of bone regeneration. Since no standardized, automated counting of immunohistochemically labeled cells is available yet it was tested that interindividual differences of cell counting between different observers did not exceed 15% of the counted cell number per visual field.

### Statistical analysis

In order to analyze cytoplasmic immunohistochemical staining and the spatial pattern of expression, the labeling index was determined as the number of positively stained cells per total cells in the visual field. Multiple measurements were pooled for each sample group prior to analysis. The data was pooled in each group as follows: 20 analyzed specimens × 3 analyzed visual fields = 60 counts of positively stained cells and 60 counts of the total number of cells; this resulted in 60 labeling indices per group. The results are expressed as the median, the interquartile range (IQR), standard deviation (SD), and range. Box plot diagrams represent the median, the interquartile range, minimum (Min), and maximum (Max). Confirmatory comparisons were performed between treatment and control groups with generalized estimating equations (GEE) that included the "treatment modality" and the "subject id" as independent factors for appropriate analysis of repeated measurements per individual. Multiple p values were adjusted according to Bonferroni by multiplying each p value obtained by the number of confirmatory tests performed (n = 10). Two-sided, adjusted p-values ≤ 0.05 were considered significant. Analyses were performed with SPSS 17.0 for Windows (SPSS Inc, Chicago, USA).

## Results

### Analysis of TGFβ1-expression

**Figure 1:**
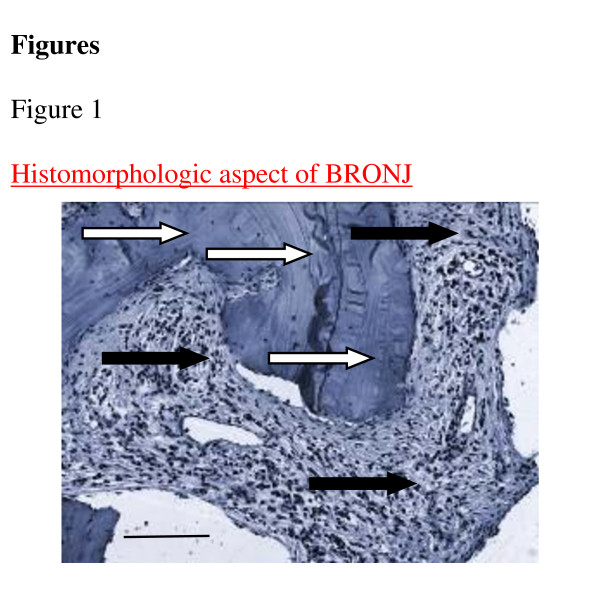
**A histopathologic section of a BRONJ-affected jaw (hematoxylin-staining, original magnification ×100) Scale bar = 100 μm**. The BP-altered bone (white arrows) shows characteristically dense bone formation, surrounded by partly inflamed mucoperiosteal soft tissue (black arrows).

**Figure 2:**
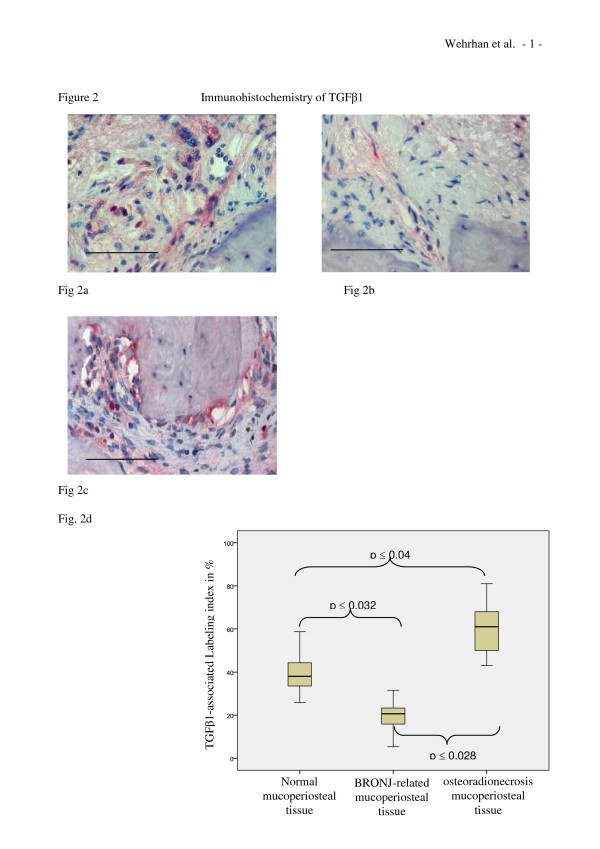
**TGFβ1 expression is reduced in BRONJ-related, but increased in osteoradionecrosis-related mucoperiosteal tissue**. (a-c) Representative immunohistochemically stained tissue sections show cytoplasmic TGFβ1 staining at × 200 magnification. Scale bars are 100 μm. (a) Immunohistochemical image showing TGFβ1 staining throughout the mucoperiosteal tissue of the jaw. Staining was distributed homogenously throughout the soft tissue. (b) Cytoplasmic staining for TGFβ1 was reduced in BRONJ-related mucoperiosteal tissue accompanied by reduced cellular density. (c) Osteoradionecrosis-related tissue showed higher stained-cell density than normal or BRONJ-related mucoperiosteal tissues. (d) The labeling index for TGFβ1 expression (Table 1) was significantly decreased (p(0.032) in BRONJ-related mucoperiosteal tissue, but significantly increased (p(0.04) in osteoradionecrosis-related tissue, compared to that for normal mucoperiosteal tissue.

The tissue sections comprised connective tissue of variable width between thickened bone formation and a layer of epithelium (Figure 1). We consistently observed partially confluent necrotic lesions in BRONJ-related bone tissue. Variable densities of inflammatory infiltrates were contained within the connective tissue layers and the ECM. Multinucleated cells were present in all BRONJ samples. Capillaries were observed in both BRONJ-related mucoperiosteal specimens and healthy jaw connective tissue. In normal jaw mucoperiosteal tissue, TGFβ1 expression was localized to the cytoplasm of fibroblasts and progenitors within the connective tissue layer (Figure 2a). The TGFβ1 was homogenously distributed within the connective tissue. In BRONJ-related tissue, a reduced cellular density of TGFβ1 expressing fibroblasts and progenitor cells was noted (Figure 2b). The connective tissue-related cells were rarely stained, and TGFβ1 expressing fibroblasts in the fibrous and inflammatory tissue surrounding the bone matrix were less dense than those observed in normal and osteoradionecrosis-related tissue (Figure 2b, c). Next, we counted the number of TGFβ1 expressing cells in the fibrous soft tissue structures, which comprised periosteal progenitors, fibroblasts, and fibrocytes, compared to the total number of connective tissue-related cells. The labeling index (ratio of TGFβ1 expressing cells/total number of fibrous tissue-related cells) was significantly diminished (p < 0.032) in the BRONJ group and significantly increased (p < 0.04) in the osteoradionecrosis group compared to the control mucoperiosteal tissue (Table 1; Figure 2d).

**Table 1:** Quantitative anlysis of immunohistochemistry results.

Protein	TGFβ1			Smad-2/3			Smad-7			Galectin-3		
*Tissue source*	*Median IQR*	*SD*	*R*	*Median IQR*	*SD*	*R*	*Median IQR*	*SD*	*R*	*Median IQR*	*SD*	*R*
Normal	38.03 11	7.92	33	35.95 8	5.73	28	40.93 14	8.93	44	15.15 7	4.8	23
BRONJ-related	20.68 8	5.24	26	17.16 8	5.22	20	62.83 15	11.3	45	44.44 20	12	45
Osteoradionecrosis-related	61.01 23	12.2	38	52 14	6.75	19	18.01 10	7.98	25	29.02 8	5.8	19

Relative labeling indices for targeted proteins among normal, BRONJ-related, and osteoradionecrosis-related mucoperiosteal tissues. Values represent the median, interquartile range (IQR), standard deviation (SD), and range (R).

BRONJ: bisphosphonate-associated osteonecrosis of the jaw

### Analysis of Smad-2/3 expression

**Figure 3:**
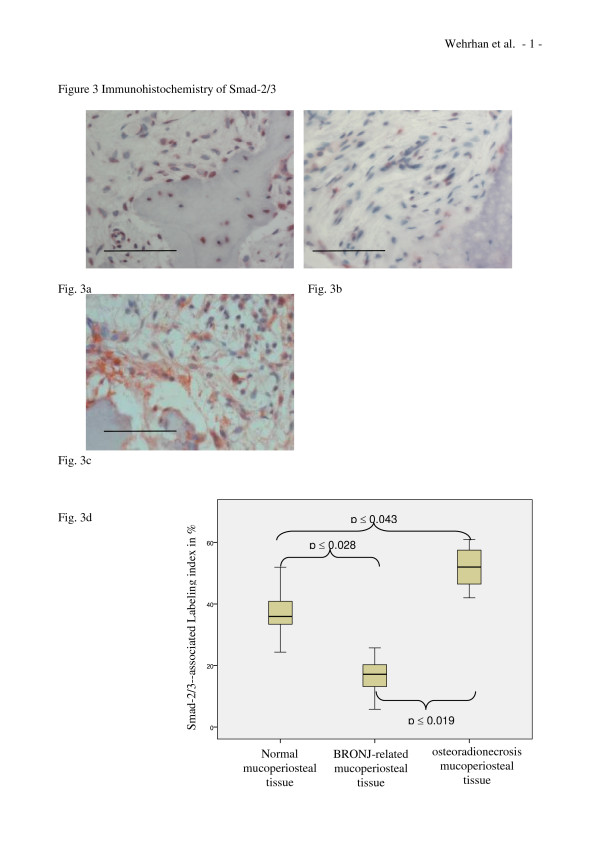
**Smad-2/3 expression is decreased in BP-altered mucoperiosteal tissue and increased in osteoradionecrosis-adjacent mucoperiosteal tissue**. (a-c) Representative immunohistochemically stained tissue sections show cytoplasmic Smad-2/3 staining at ×200 magnification. Scale bars are 100 μm. (a) Ubiquitous Smad-2/3-staining was observed in healthy mucoperiosteal tissue; (b) decreased Smad-2/3 staining was found in BP-altered BRONJ-related oral mucoperiosteal tissue. (c) Osteoradionecrosis-related soft tissue Smad-2/3 expression was increased compared to controls. (d) The labeling index of Smad-2/3 expression (Table 1) was significantly decreased (p(0.028) in the BRONJ-related mucoperiosteal tissue and significantly increased (p(0.043) in osteoradionecrosis-related tissue compared to that in normal mucoperiosteal tissue.

Smad-2/3 expression was observed in the samples of healthy jaw mucoperiosteal tissue (Figure 3a), in BRONJ tissues (Figure 3b), and in osteoradionecrosis-adjacent soft tissues. The densities of Smad-2/3 expressing fibroblasts, fibrocytes, and periosteal progenitors were reduced in the BRONJ group compared to healthy group. Periosteum and connective tissue cells predominantly exhibited nuclear Smad-2/3-staining in all groups. The median labeling index of Smad-2/3 expressing fibroblasts, fibrocytes, and periosteal cells was significantly reduced in the BRONJ-related (p < 0.028) compared to control mucoperiosteal tissues (Table 1 Figure 2d). In the osteoradionecrosis-related group (Table 1 Figure 3d), the labeling index indicated significantly increased cellular Smad-2/3 expression (p < 0.043).

### Analysis of Smad-7 expression

**Figure 4:**
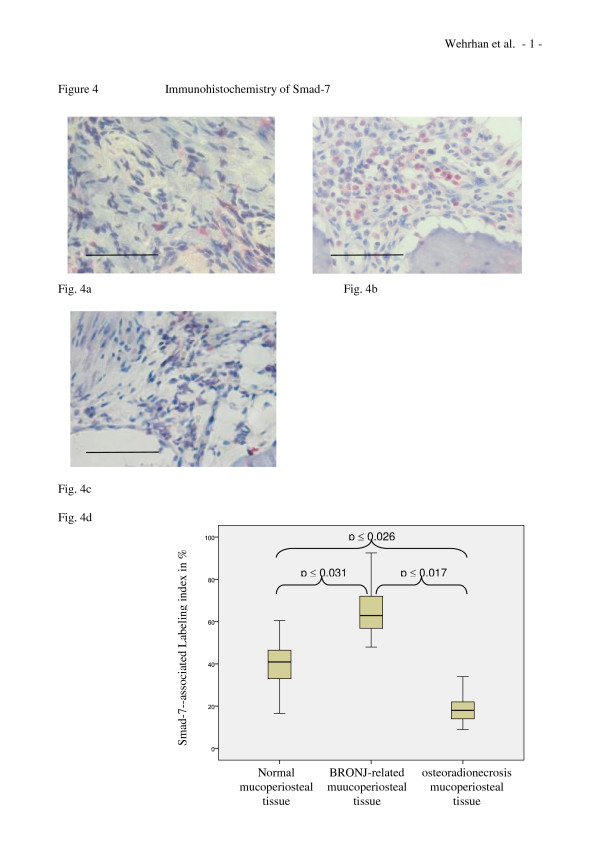
**The expression of TGFβ1-inhibiting Smad-7 is upregulated in BRONJ-adjacent mucoperiosteal tissue, but decreased in osteoradionecrosis-adjacent oral mucosa**. (a-c) Representative immunohistochemically stained tissue sections show cytoplasmic Smad-7 staining at × 200 magnification. Scale bars are 100 μm. (a) Cellular Smad-7 expression was only rarely detected throughout healthy mucoperiosteal soft tissue. (b) An increased number of cells with Smad-7 positive staining was observed in BRONJ-related mucoperiosteal tissue. (c) Osteoradionecrosis-related mucoperiosteal tissue showed increased expression of Smad-7 compared to that observed in BRONJ-related tissue and controls. (d) The relative number (labeling index) of Smad-7-expressing cells was significantly increased in BRONJ samples (p(0.031) (Table 1).

The pattern of Smad-7 expression differed between the specimens from normal (Figure 4a), BRONJ-associated, (Figure 4b) and the osteoradionecrosis-related samples (Figure 4c). Compared to the soft tissue of normal jaw samples, nuclear Smad-7 expression was increased in BRONJ periosteal soft tissue cells. However, BRONJ samples showed inhomogeneous spatial distributions of Smad-7 expressing cells in soft tissues; the highest density was detected at the periosteal margins attached to the bone structures. In contrast, osteoradionecrosis-related mucoperiosteal tissue showed only a few Smad-7-stained cells (Figure 4c). Thus, compared to control tissues, the overall density of Smad-7-expressing cells was significantly increased in BRONJ tissue (p < 0.031) (Table 1 Figure 4d) and significantly decreased (p < 0.026) in the osteoradionecrosis-related tissue (Table 1 Figure 4d).

### Analysis of Galectin-3 expression

**Figure 5:**
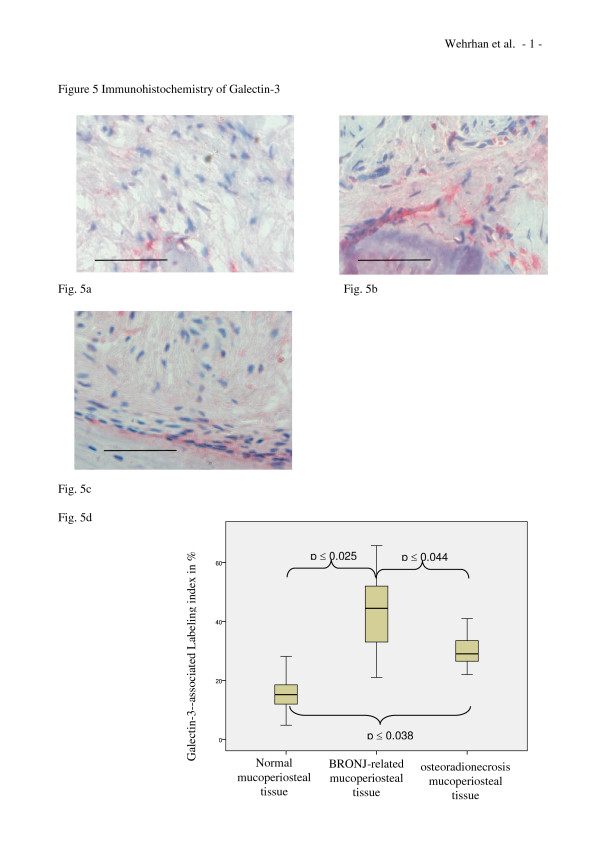
**Galectin-3 expression is increased in BRONJ-affected and osteoradionecrosis-related mucoperiosteal tissues**. (a-c) Representative immunohistochemically stained tissue sections show cytoplasmic Smad-7 staining at × 200 magnification. Scale bars are 100 μm. (a) Expression of Galectin-3 in healthy mucoperiosteal tissue was restricted to the periosteal margin and cells adjacent to the bone-soft tissue interface. (b) Galectin-3 expression in the BRONJ-affected mucoperiosteal tissue was distributed throughout the entire soft tissue. (c) Osteoradionecrosis-related mucoperiosteal tissue also showed Galectin-3 staining. (d) The relative number (labeling index) of Galectin-3-expressing cells was significantly increased in BRONJ (p(0.025) and osteoradionecrosis samples (p(0.038) compared to control (Table 1).

Galectin-3 was detected in the periosteum and the overlying periodontal tissue layers of healthy jaw tissue samples. The cytoplasmic staining pattern in normal tissues was different than the patterns found in BRONJ and osteoradionecrosis-associated tissues. In normal jaw tissues, Galectin-3 staining was concentrated in the periosteal cell layers (Figure 5a). In contrast, BRONJ-related jaw soft tissue (Figure 5b) and osteoradionecrosis-adjacent tissue (Figure 5c) showed Galectin-3 staining throughout the tissue samples (Figure 5b, c). Homogenous cytoplasmic Galectin-3 staining was observed in the fibrous tissue stroma cells between the periosteum and the epithelium of the oral mucosa in BRONJ-affected and osteoradionecrosis-related soft tissue. In contrast, only selective staining was observed in the fibrous tissues of the normal jaw. The overall cellular density of Galectin-3-expressing cells was significantly increased in the BRONJ (p < 0.025) and osteoradionecrosis-adjacent tissues (p < 0.038) compared to the periosteal fibrous tissue of the normal jaw.

## Discussion

This was the first study to address the influence of BP on key regulators of oral mucosa tissue regeneration in BRONJ. We found that BRONJ-affected mucoperiosteal tissue showed significantly diminished expression of the pleiotropic growth factor TGFβ1 (p < 0.032) compared to controls. Moreover, TGFβ1-related intracellular signaling through Smad-2/3 was significantly decreased (p < 0.028), and TGFβ1-inhibition through Smad-7 was significantly increased (p < 0.031) in BRONJ compared to controls. The expression of glycoprotein Galectin-3, known to be a differentiation marker for osteoblasts and chondrocytes, was significantly increased (p < 0.025) in the BRONJ-adjacent oral mucosa soft tissue [14] compared to controls. The reduced expression of TGFβ1 in BRONJ-related tissues is associated with a diminishment in collagen I- and -III expression and reduced stimulation of ECM components {Wehrhan, 2004 #3275;Schultze-Mosgau, 2006 #2686}.

This abrogated TGFβ1-signaling was substantiated by the concomitant decreased expression of Smad-2/3. This result suggested that BRONJ caused the suppression of both the growth factor TGFβ1 and its downstream signaling pathway. The loss of TGFβ1-related cellular activation in BRONJ-affected oral mucosa connective tissue could explain the prolonged wound healing and the lack of mucosal regeneration in BRONJ lesions. Indeed, this study confirmed the *in-vitro *finding that collagens I and III expression decreased in oral mucosa fibroblasts following application of zoledronic acid [7]. Our results suggested that *in-vivo *stimulation of ECM protein deposition would most likely be inhibited, due to the increased expression of Smad-7, which inhibits TGFβ1-activity. In contrast to skin and mucosa fibrosis, which is characterized by excessive expression of TGFβ1 and Smad-2/3, accompanied by suppression of Smad-7, the BRONJ-affected tissues were in a sclerotic state brought about by the imbalance in TGFβ1 signaling [1,23]. The findings of this study provided evidence that the etiopathological development of BRONJ is different from other diseases that present exposed jaw bone. For example, osteoradionecrosis has been shown to be associated with increased expression of TGFβ1 [23]. This study showed that BRONJ-adjacent soft tissue and osteoradionecrosis-related mucoperiosteal tissue had differential impairments in TGFβ1-related signaling. Osteoradionecrosis-affected tissues showed upregulation of TGFβ1 and Smad-2/3 expression and suppression of Smad-7; this was the opposite of findings in BRONJ-affected tissues.

Oral mucosa morphology features a direct hemidesmosomal connection between the periosteum and the basal lamina. This implies that connective tissue fibroblasts originate from periosteal progenitors [24]. Therefore, BP-related transdifferentiation of oral periosteal progenitor cells would be expected to influence the cellular identity and proliferation of periodontal tissue stromal cells. This suggestion was supported by the recent finding that Msx-1 expression was reduced in BP-exposed periosteum [3]. Moreover, impairment of the TGFβ1-driven EMT in BRONJ sites led to both reduced re-epithelization of the wound surface and altered differentiation of connective tissue progenitors {Vincent, 2009 #3998}. In osteoradionecrosis-related mucoperiosteal tissues, the overexpression of TGFβ1 causes an arrest of the EMT process in activated myofibroblasts; conversely, in BRONJ, the lack of TGFβ1 and Smad-2/3 activity attenuated the stimulation of EMT {Schultze-Mosgau, 2004 #2678}.

In addition to suppressing cell proliferation, BPs have been shown *in-vitro *to induce osseous differentiation in periosteal cells [6]. Furthermore, BPs have been shown to enhance recruitment and differentiation of osseous progenitors in the periodontal ligamentum [25]. Those findings suggested that a reduction of connective tissue differentiation and increased osseous stimulation are likely to occur during jaw periosteal and periodontal progenitor cell differentiation [26]. The BP-induced alteration in connective tissue cell differentiation was reflected by the increased expression of Galectin-3 in periosteal progenitors in BRONJ tissue. Galectin-3 is involved in the differentiation of osteoblasts and chondroblasts [27]. Increased Galectin-3 levels have been shown to inhibit epithelial cell proliferation {Szabo, 2009 #4519}. In BRONJ-related tissues, in the absence of TGFβ1-stimulation, Galectin-3 was associated with osteogenic cell differentiation. In osteoradionecrosis-related mucoperiosteal tissues, increased TGFβ1 and Smad-2/3, together with radiation-induced Galectin-3 were associated with perpetuation of fibrotic soft tissue remodeling and inhibition of re-epithelization {Cao, 2002 #4522}. During bone development, Galectin-3 is expressed up to the stage of hypertrophic chondrocyte formation, but it is downregulated in osteoblasts and osteocytes undergoing terminal osseous differentiation [28]. Induction of Galectin-3 expression and increased cellular recruitment of Galectin-3 in BRONJ-related oral mucosa tissues reflected BP-associated progenitor cell transdifferentiation towards an osteogenic phenotype [25]. These cellular biology results are consistent with the very recent notion that an aseptic alveolar bone alteration may be the key mechanism underlying the development of BRONJ [29]. One study described initial cellular and morphological osteopetrotic changes in the bone matrix prior to the clinical appearance of BRONJ [29]. Radiographic signs of osteopetrotic jaw bone architecture due to BP-therapy have been demonstrated in the absence of BRONJ {Reid, 2009 #3902}. Therefore, soft tissue lesions appear to reflect a secondary phenomenon during the development of BRONJ. HIF-1 α and hypoxia are known to induce Galectin-3-mediated osteoblast survival. Thus, following laceration of the BP-altered periodontal tissue, the ensuing tissue hypoxia could be expected to increase osseous stimulation of progenitor cells and enhance the ongoing suppression of connective progenitor cell proliferation {Riddle, 2009 #4005}. The clinical observation of painless, exposed jaw bone and non-reactive mucoperiosteal tissue in BRONJ tissues might be explained by the increased Galectin-3, which is known to mediate inhibition of intraoral inflammation [1,16]. Galectin-3 was shown to specifically inhibit LPS-associated cytokine activation, a characteristic of intraoral gram negative bacteria [16]. The potential role of Galectin-3 in preventing an intraoral immune response in BRONJ is further substantiated by the significantly higher expression (p(0.044) of Galectin 3 in BRONJ-affected tissue compared to osteoradionecrosis-related tissue, which is characterized by local inflammation. Finally, BRONJ-affected tissues exhibit anergy; this was shown to be successfully prevented in therapeutic approaches that prevented salivary contamination following surgical BRONJ-sequestrectomy [18]. This supported the notion that infection is not regularly associated with BRONJ.

In conclusion, this study was the first to investigate impairments in the signal transduction pathway related to oral mucosa soft tissue repair in BRONJ. Our findings indicated that BRONJ was associated with an impairment in TGFβ1 signaling that was different than that associated with osteoradionecrosis of the jaw. As recommended by leading international experts in the field of BRONJ, we have shown that targeting morphological and cellular features unique to the mucoperiosteal tissue was a promising approach for elucidating the pathologic mechanisms underlying BRONJ [1]. To our knowledge, this is the first study to describe the differences between BRONJ-affected, osteoradionecrosis-related, and healthy oral mucosa tissues in the TGFβ1 signaling pathway. These findings revealed that the mechanisms underlying the development of BRONJ involved an aseptic, osteopetrotic alteration in the jaw bone, followed by a secondary reduction in the regeneration capacity and a specific reaction in mucoperiosteal soft tissue.

## Funding statement

This study was funded by the ELAN-Fonds of the University of Erlangen-Nuremberg, Germany.

## Competing interests

The authors declare that they have no competing interests.

## Authors' contributions

The authors' initials are used.

FW applied for grant support (ELAN-Fonds, University of Erlangen), initiated and conducted the study, formulated the hypothesis, established and conducted the methods and analytic procedures, and wrote the manuscript. PH formulated the hypothesis and interpreted the data. AG performed the histomorphologic analysis of the changes in BRONJ-affected oral mucosa and mucoperiosteal soft tissue. PS and KS performed the immunohistochemical analysis.

FN interpreted the data and wrote part of the manuscript, particularly the discussion section. EN interpreted the data and harvested the samples. KA established the immunohistochemistry, analyzed the tissue samples, interpreted the data, and performed the histopatholgic analysis of BRONJ-related and control tissue samples. All authors read and approved the final manuscript.
